# Autonomy and Authority in Public Research Organisations: Structure and Funding Factors

**DOI:** 10.1007/s11024-018-9349-1

**Published:** 2018-03-03

**Authors:** Laura Cruz-Castro, Luis Sanz-Menéndez

**Affiliations:** Institute of Public Goods and Policies (IPP), Consejo Superior de Investigaciones Científicas (CSIC), C/ Albasanz 26-28, 3D, 28037 Madrid, Spain

**Keywords:** Public research organisations, Research funding, Professional organisations, Authority relationships, Autonomy of researchers, Organisational typologies

## Abstract

This paper establishes a structural typology of the organisational configurations of public research organisations which vary in their relative internal sharing of authority between researchers and managers; we distinguish between autonomous, heteronomous and managed research organisations. We assume that there are at least two sources of legitimate authority within research organisations, one derived from formal hierarchy (organisational leadership) and another derived from the research community (professional); the balance of authority between researchers and managers is essentially structural but is empirically mediated by the funding portfolio of organisations and the corresponding endowment of resources at the disposal of leaders or researchers. Changes in the level, sources and strings of organisational and individual research funding are expected to affect the balance of internal authority in different ways depending on the organisational configuration, and to open the door to the influence of external actors in the development of research agendas.

## Introduction

In recent years Public Sector Research (PSR) has undergone important changes. Universities have gradually become the major actors undertaking research in almost all European public research systems (Paradeise et al. 2009; Nedeva 2013), but non-university Public Research Organisations (PROs) continue to play an important role in many countries (Larédo and Mustar 2004). The growth of some R&D systems has occurred simultaneously with the creation or expansion of new types of research performing organisations (Gulbrandsen 2011), the increase in the diversity of missions and activities undertaken by existing research organisations and their reform and mergers. In comparison with higher education institutions, the non-university public research organisations have received little research attention;^1^ this paper aims precisely at providing some insights for their analysis.

Two dynamics are worth noting. Firstly, in some countries demands for accountability from governments and the implementation of reforms inspired in New Public Management (NPM) have favoured an increase in the autonomy of PROs, and in universities, in relation to the state, and have granted managers or administrators more formal authority over resource allocation and personnel decisions to meet the research targets set by agencies (Schimank 2005; Musselin 2006; Whitley and Gläser 2014). Pressures for the adaptation of PROs have also increased; in times of recession many kinds of research organisations search for external funding (Sanz-Menéndez and Cruz-Castro 2003), reducing the steering capability of their direct funders and leading to a higher level of competition for the limited resources available. Additionally, R&D budget cuts and expenditure controls may reduce the autonomy of organisations (Cruz-Castro and Sanz-Menéndez 2016).

Secondly, there have been changes in the way governments develop research policies and fund PROs of various types. Three major trends have been identified: firstly, a changing balance between block and project funding (Van Steen 2012); secondly, a transition to performance-based block funding mechanisms in universities and other research organisations (OECD 2010; Hicks 2012; Whitley 2008); and thirdly, an increasing role for industry funding in public research institutions (Slaughter and Leslie 1997; Behrens and Gray 2001; Hottenrott and Lawson 2014). However, it remains true that research organisations themselves continue to be an important channel of funding for individual research.

All such changes in the funding of academic research have been argued to have consequences for the dynamics of science and for the autonomy of researchers in the pursuit of their own curiosity-driven research agendas (Ziman 2000), for the cognitive development of science (Braun 1998) or for the promotion of scientific innovations (Whitley 2014). The issue of the increasing limitations on the autonomy of researchers in their choice of topics and methods has also been addressed in connection with the impact of bureaucratisation in research (Walsh and Lee 2015).

One of the most important channels of influence over research agendas is through the allocation of resources and funding; the issues of patrons and sponsors of research (Turner 1990) and tension with researchers’ autonomy (Cozzens 1990) have been recurrent in studies of scientific research. However, in a recent review, Gläser and Laudel (2016) have shown that little is known about the impact of the transitions to split funding models on research content. At best, findings are ambiguous and there are methodological difficulties for causal attribution of macro level changes in research to changes in governance instruments; these authors suggest the need to address the macro-micro links. We believe that such links are framed by structural organisational attributes which mediate relations of researchers with funders. Today “research is a professional, intellectual activity performed in diverse organisational settings” (Lambright and Teich 1981: 316); organisations may have their own collective goals. We draw on insights from the theory of professional organisations to account for variations in the ability of researchers to set their own agendas.

We assume that there are at least two sources of legitimate authority within research organisations, one derived from formal hierarchy (bureaucratic leadership) and another stemming from the community (professional), and that the balance of authority between researchers and managers is essentially structural but will be empirically mediated by the dominant funding portfolio of organisations and the corresponding endowment of resources at the disposal of leadership or researchers. Control of knowledge and its application are key elements of the professional nature of research, but we also think that in certain organisational structures researchers share to varying degrees their authority over the work process and research content with other actors.

The aim of the paper is twofold; firstly, to identify different structural conditions and their connection with the autonomy of researchers to pursue their research goals, with the aim of characterizing certain types of organisational research configurations. Secondly, we aim to incorporate the funding dimension (in an exploratory way) into a more comprehensive framework that focuses primarily on the internal authority balance between researchers and managers, but also takes into account how different types of funding affect that balance.

In the next section we revise the concepts of authority and autonomy, stressing their relational nature, the idea of authority sharing and the concept and types of professional organisations. In the third section we address the question of how organisational structures affect the relative power of different groups within research organisations and distinguish between three configurations. The fourth section describes how different types of research funding can influence control in research organisations, and develops the analytical typology of organisational configurations, centred on authority sharing. For each of the structural configurations described, we also advance some propositions about how changes in funding types are likely to affect the level of researchers’ autonomy and the sharing of authority. We conclude by advancing some ideas about the role of the relative wealth of organisations and resource intensity in different fields.^2^

## Authority Sharing Over Research Objectives and Content

Macro analyses of the changes in research activity and the research system in recent decades have described them as changes in the governance of science (Borrás 2012). The underlying idea of the governance framework is that decision-making processes are shaped and influenced by more actors than are formally or legally established. Gläser (2010) and Whitley (2008), in an attempt to overcome the extremely wide and diffuse meaning of governance, have proposed the concept of authority sharing as a conceptual device that provides a specific focus for the different roles of actors in shaping research activities and agendas.

In their perspective, authority refers to the legitimate power of actors; an actor’s authority is defined as institutionally shaped influence (Gläser et al. 2014: 301), and authority relations mean the relative authority of a set of independent actors. One of the advantages of the notion of authority sharing is that it can be specified in relation to specific decision-making processes (Gläser 2010: 358). One of their principal arguments has been that researchers have had to increasingly share authority over research decisions with other actors who provide access to funding and affect researchers’ conditions for making a reputation and advancing their careers. Incorporating the search for funding into the credibility cycle (Latour and Woolgar 1979/1986; Rip 1994) allows external actors to shape research agendas. Perhaps the transformational nature of these changes to governance and shifts in authority has been overstated,^3^ because research enterprises have always needed funding and sponsors, and researchers have always depended on other actors for accessing funds, recognition and promotion.

Underlying the argument concerning changes is a dominant normative view that the shift away from researchers in the relative authority of different groups and organisations over research goals has a negative impact on innovation, creativity and change in science (Geuna et al. 2003). However, Whitley (2011: 381–382) has acknowledged that the combination of different shifts and trends could have contradictory effects and that while some changes may narrow the scope of researchers’ discretion over research goals, others could mitigate such consequences, depending on the context in which they are introduced; nevertheless, the role of organisational structures has been underestimated in these approaches.

### Authority and Autonomy in Research Organisations: Discretion and Work Control

Organisation theory has usually established differences between authority and power and has mainly addressed authority as one form of power (Weber 1947; Blau and Scott 1962: 27). Power, in general terms, involves a relationship between two or more actors in which the behaviour of one is affected by the behaviour of others. Power is a relational variable: isolated individuals cannot have power.

Authority may be seen as “the power to make decisions which guides the actions of another” (Simon 1951). Authority is a type of power that “is based on the acceptance by others of a given individual’s legitimate right to issue orders or directives” (Weber 1947; Hall and Tolbert 2005: 88); it requires a common value system among the members of the collective. Authority could involve “interpersonal” relations or “inter-unit” relations (e.g. between hierarchical levels, or within the same levels of different departments). In addition, even social actors who are not internal members of the organisation could influence others inside it to behave in particular ways.

Aghion and Tirole (1997) distinguish between formal authority, or the right to decide, and real authority, which is the effective control over decisions. A formally integrated structure in research organisations can accommodate various levels of real integration/delegation. Real authority is determined by the structure of information, in the sense of having the necessary expertise; when the principal or the manager is informed, it is easier for real authority to prevail. A related distinction refers to the difference between positional and relational authority. The latter concept is of special interest in the analysis of professional authority. Whereas positional authority is essentially structural, relational authority could be defined as the capacity to elicit voluntary compliance based on transactions and it introduces a dynamic dimension into authority relations. The bases of such transactions could be resources, including expert knowledge. Our approach in this paper acknowledges the interplay between structures and processes, between positions and relations.

Autonomy, for professionals who are employees in any organisation, is necessarily limited, but one of its attributes, discretion, enables professionals to assess cases and decide on action (Evetts 2002: 345). Of course, in the exercise of discretion professionals take into account all factors and requirements in a given context (organisational, economic, etc.) (Freidson 2001: 34–35). In fact, this process of interiorising the organisational context of research and the power of other actors in relation to research agendas has been already well documented (e.g. Knorr-Cetina 1981). Together with discretion, a second relevant factor that characterises professionals and researchers is the idea of “control” of work (Tolbert 2004). For the purpose of this paper, we use the concept of the “autonomy” of the researcher as discretion over the goals and/or methods and control over the work process.

Theoretically, there is no reason to assume a conflict between individual autonomy and organisational goals. An interesting distinction (Bailyn 1985) refers to “strategic autonomy”, the freedom to set one’s own research agenda and directions versus “operational autonomy”, the freedom, once the problem has been set, to tackle it by particular means, this is, the discretion to decide how to pursue that goal. Work in research organisations requires both strategic and operational controls, yet these could be allocated to the same or to different actors. Additionally, different forms of autonomy might be unevenly distributed among researchers depending on their position in the social structure of the organisation and the profession (tenure, rank, reputation, etc.). In this sense, coming back to the idea of relational authority, it is important to take into account the social positions that researchers occupy in one or more structures or networks, including the scientific community and the clients (research funders), in which they develop relations providing them with different types of resources.

### Professional Organisations

Professional organisations have been defined as “organisations in which members of one or more professional groups play a central role in the achievement of the primary organisational objectives” (Scott 1965: 65–66). Based on “the amount of autonomy granted to professionals by the administrative controls structure” he originally defined two types of professional organisations: *autonomous* and *heteronomous*. In the *autonomous* type, the organisational leadership delegated to the professional group of employees “considerable responsibility for defining and implementing the goals; for setting the performance standards, and for seeing to it that standards are maintained”. A clear demarcation between professional and administrative officials’ responsibilities, jurisdictions and zones of control was expected; another hallmark is that the dominant professional group organises itself as a professional staff to support and monitor the performance of its members, with the seniority principle (tenure) operating as an important basis of control. Examples included hospitals, universities and scientific institutes oriented to basic research.

In the *heteronomous* organisation type, professional employees are subordinated to an administrative framework and their amount of autonomy is limited by the presence of systems of supervision and control of the tasks performed by the professionals; examples included secondary schools, social welfare agencies or firms engaged in applied research. In contrast with the autonomous type, there are no sharp distinctions between the professional and administrative spheres of action. Although this type of organisation may appear to be conventional bureaucratic hierarchies, there are important differences between the heteronomous organisations and bureaucratic hierarchies regarding the autonomy of professionals. In most cases, managers are themselves professionals.

Scott (1982), examining different forms of managing professional work in hospitals, introduced a new organisational type: the *conjoint professional organisation,* in which professional participants and administrators are equal in power and coexist in a state of interdependence and mutual influence. Interestingly enough, Scott cited research organisations as examples of this type of structure, with multiple centres of power and pluralistic relations, tending towards a balance between the conditions conducive to creativity and the conditions conducive to control. The basic distinction here is between the concerns of professionals regarding projects and the focus of administrators and managers on the global or macro concerns of the organisation.

Positioning professional organisations in the wider context of different types of organisations, Mintzberg (1993: 189) characterised *professional bureaucracies,* based on the idea that organisations may be bureaucratic without being centralised. The coordination of relatively independent professionals is achieved through the standardisation of skills (conferred through professional training) and internalised values, rather than through formalised systems and close supervision. The standards of the professional bureaucracy originate largely outside its own structure. The decision-making structure of the professional bureaucracy reflects collegiate values and, in general, professionals seek collective control over the administrative decisions that affect their operations. Funders, as a type of client, are essential in professional bureaucracies, yet there is considerable evidence that this model has undergone significant change. In particular, professional public sector organisations have changed considerably since the 1980s, with the introduction of NPM principles (performance, cost efficiency and audit oriented).^4^ We believe, however, that this classic typology is a sound basis for our paper because in the construction of the structural configurations we have worked mainly at the level of general, ideal types.^5^

It has long been recognised that in organisations where the staff is highly specialised or professionalised, hierarchical authority is less effective than professional one. Scientific research in organisations has represented a canonical case in which administrative authority from above is often challenged by collegial professional authority from below (Pelz and Andrews 1966). While the classical papers expressed the inherent conflict between the two sources of authority, further evidence made it clear that organisations adapt to professions and vice versa (Kornhauser 1962; Marcson 1962). Different types of organisation present different balances between the two types of authority. Furthermore, a marked tendency in the recent literature on professional organisations has been to argue that conflict between managers and professionals has been overstated (Noordegraaf 2016). Managers and professionals have found ways to link their domains through, for instance, the introduction of dual management systems, or instead to protect their respective spheres and “buffer” each other’s external influences (Noordegraaf and van der Meulen 2008; Huising 2015). This branch of literature has evidenced such processes of mutual adaptation and connectivity in the public sector. Additionally, professionals can be managing professionals, or professional and organisational logics can be intertwined (Clarke and Newman 1997), a tendency that some scholars have termed as “hybridized professionalism” and “hybrid professional/managerial roles” (Noordegraaf 2007).

## Research Organisations as Professional Organisations: Structural Configurations

We now take the three structural types of professional organisations and propose a classification of research organisations based on certain basic attributes (Table 1). Two clarifications are necessary at this point. Firstly, our goal is not empirical; we do not aim to classify existing public research organisations into various categories (that would involve the construction of a taxonomy and this is not our aim here) but rather to describe the properties of different systems of coordination, supervision and control that lead to different organisational configurations. We are less interested in formal real organisations in general than in “forms of organising”. However, some cases can be mentioned, to situate the reader in the existing landscape of research organisations; such illustrations should be taken more as potential cases for the empirical testing of the value of the typology than as actual examples of each categorical type.

**Table 1:** General features of the different types of public research organisations

	Research organisation types as professional organisations		
	*Autonomous* Research Organisations	*Heteronomous* Research Organisations	*Managed* Research Organisations
*Goals*			
Organisational goals	Diffuse, ambiguous and generic; limited, mainly related to tasks	Specific, clear and confined	Specific but broad, established by management
*Control systems*			
Strategic control systems	Consensus decision-making	Directive decision-making	Directive decision-making
Financial control systems	Limited financial and competitive targets, usually short term	Clear financial and competitive targets	Clear financial and competitive targets, usually long term
Operational control systems	Academic professional standards, quality and reputation	Related to the task definitions and activities planning	Professional standards, quality and outcomes, more planning
*Structure*			
Structure differentiation	Low levels of specialisation and dominant personal interests	High levels of specialisation and functional differentiation	Medium levels of specialisation and increasing functional differentiation
Structure integration	Limited use of formal rules and procedures, dominant role of professional quality and standards	Dominant role of formal rules and procedures and hierarchies	More emphasis on professional standards and quality, but also of general rules, dual hierarchical structures

Secondly, we focus on public research organisations,^6^ which themselves show considerable variation, and not on private firms. In Table 1 we identify the structural properties of different research organisational configurations of public research entities and group them around objectives, control systems and differentiation versus integration properties.

In Autonomous Research Organisations, the collective goals are quite generic or diffuse; in essence the organisational goals will be no different than the development of the tasks, namely conducting and communicating research. Organisational outputs will be largely the aggregation of individual contributions, with little coordination. The overall structure is quite decentralised and the distinction between the administrative and the scientific spheres of action rather sharp. Among researchers, the level of specialisation in particular tasks is quite low in the sense that they master their field in broad terms and are expected to conduct all phases of research, from problem choice to the diffusion of results. Structural differentiation in these organisations is based on scientific field divisions and personal interests. To provide the reader with cases of existing organisations in the public research field we would expect to fit in this category, we could mention national academies of science or national research councils, like the CNRS in France, the CONICET in Argentina, the CNR in Italy, the CSIC in Spain, etc. They largely represent the classical model of academic science organisation, similar to research units in universities, but without the formal role or mission of teaching.

Heteronomous Research Organisations have specific goals and their management is capable of establishing a top-down organisational strategy to fulfil those goals; appointed managers or directors have mandates either from political authorities or from their Boards. Coherently with directive decision-making, strategic planning and financial control become separate organisational functions. There is a substantial degree of professional specialisation compared with the more generalist nature of scientists in autonomous ones; their structures display greater horizontal division, and the criteria for differentiation is related to functional differences rather than to scientific fields or interests.

The relatively large administrative component helps to resolve problems of coordination and the integration of contributions. In line with the increased division of labour and the standardisation of tasks, the advanced skills of performers and the need to coordinate individual contributions in order to accomplish specific goals, such organisations augment their hierarchy with more managers with a reduced span of control. The operational structural form in this type of research entity is likely to be departments, organised consistently with the dominant hierarchical authority principle, in which heads of department are responsible for the work of their members. Regarding empirical cases that share these attributes to a greater or lesser extent, there can be mentioned the field or mission-specific research centres embedded in the structures of the public administration in the areas of health, energy and environment, agriculture, defence, etc. that exist in many countries: National research centres specialised in agriculture (INRA—France, INTA—Argentina), defence and aerospace (NASA—US), energy and the environment (NREL—US; CERI—Canada, CIEMAT—Spain), health (INSERM—France, INSA—Portugal), or some diversified centres with the general mission of promoting industrial competitiveness (TNO—Netherlands; VTT Finland; Tecnalia—Spain) are expected to be under this type.

Managed Research Organisations display a balance between professional and administrative controls^7^ and exhibit features that fall between the other two types. Such organisations integrate the concern for coordination and strategic collective action in their functioning with more informal but hierarchical structures where we would expect to find research groups organised around programmes, including one or various projects, with a scientific leader or supervisor playing the role of Principal Investigator (PI).^8^ In between the other two types, managed research organisations are characterised by medium levels of specialisation and functional differentiation, with decision-making more directive than consensus based, but mixing top-down and bottom-up mechanisms. Empirical cases expected to fit into this category are likely to show great heterogeneity. It is possible to think of some research and technology organisations, some fundamental research institutes with strong directorship’s role (like the MPG in Germany), new research institutes (like CNIO, IRG, etc. in Spain or INMEGEN in Mexico), or organisational innovations from more academic settings, such as centres of excellence or other hybrid forms.

## Research Funding and Authority Sharing

In the previous section we presented the main features of the three structural types of professional forms of organisation (autonomous, heteronomous and managed). In this section we further develop the framework to specify the features of each type in relation to authority sharing and the autonomy of researchers, and put forward some propositions about how different types of funding might affect such dimensions. Before further exploring the typology, we first explain the ways in which public research organisations and researchers may be funded and the expected effects on authority sharing.

### Funding Resources and Control in Research Organisations

Authority relations and intra-organisational autonomy do not only derive from structure. Resource dependency theorists have shown that the power of organisational actors is contingent on their control of critical resources. From this perspective, changes in the structure of the resource environment influence the relative authority of the leadership vs. professionals.

Some previous literature related to the impact of funding on research agendas and organisations has either considered the relationship between funders and researchers (e.g. Braun 1998) as if the researcher was not part of an organisation or, when considering the relations of funders and organisations, has argued that funding modalities determine organisational types (e.g. Wilts 2000), a claim that we do not share.

Research activity is always performed by researchers, but the resources and funding for such research may be internal and reach researchers directly through the organisation, or be external and proceed from public or private sources. For the sake of simplicity, we assume that the activity of the researcher (and his/her position) could be financed either by his/her employing organisation’s resources or by third party funders (public or private). Funders do not only fund individuals (principal investigators) but also provide resources to “public” and other types of research organisations through so-called institutional or organisational funding, which may have different types of strings attached. For the purpose of our analysis we distinguish between organisational funding and individual researcher funding. Although this distinction may recall the difference between institutional and project funding (Lepori et al. 2007), this identification could be misleading because it mixes funding targets with funding instruments.

The overall level of recurrent research funding of an organisation (what others have termed research block grants) determines its resource dependence and interacts with its structural attributes, affecting and in some cases modifying internal authority structures. The key difference is whether funding instruments target the organisation or the researcher. Funding instruments targeted on the organisation (programme funding, performance based funding schemes, organisational excellence programmes, etc.) may provide the leadership of research organisations with important resources. But it is also important to consider the strings with which funding reaches the organisation.

Considering only funding proceeding from government, there can be, to simplify, two types of organisational funding, usually called block grant: earmarked and discretionary. In some research organisations, the bulk of earmarked funding is for basic operational costs and the biggest share is usually reserved to pay the salaries of permanent researchers. By definition, earmarked funds, due to their reserved nature, allow little room for manoeuvre regarding their use; managers of organisations receiving predominantly this type of funding are likely to have less influence over the strategic research agenda than directors receiving greater shares of discretionary research funds.

By contrast, it can be stated, in general, that organisational funding which reaches a research organisation without strings might be a very powerful mechanism in the hands of the managerial leadership to influence the direction of research programmes and the decisions of researchers regarding agendas.^9^ Discretion in the use of such funds affords managers more authority, but at the same time they become more accountable to funders as collective representatives of the organisation. Some funding instruments will make organisations compete for this type of funding (e.g. performance-based schemes); others will involve organisations being held accountable for the accomplishment of science and development objectives (e.g. programme funding). The growing importance of this kind of policy instruments in many European research systems is in line with the policy rationales of granting research institutions more autonomy in their operations and, occasionally, providing managers with more leverage.

Public Research Organisations may also be funded by industry in the form of contracts or donations. This type of funding is usually linked to specific projects, programmes or services and the room for manoeuvre of the leadership concerning those resources will depend on the existing control structure of the organisation.

Turning to individual funding, researchers who are capable of receiving and controlling external funding resources on an individual professional basis may not only see their autonomy *vis à vis* the leadership increased, but they may be also capable, through professional collective action, of pushing management toward organisational strategies that benefit their interests regarding recruitment, resources for their field, department etc.

**Fig. 1:**
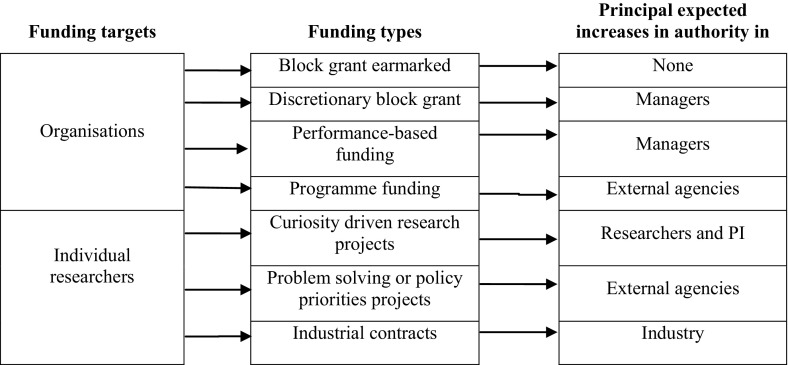
Expected effects of different types of funding on increases in actors’ authority over research goals (without considering any organisational form)

It can be said that in general terms individual funding reinforces the internal authority of researchers versus managers and therefore the autonomy of the former (see Fig. 1). However, it is possible to identify at least three forms of individual researcher third party funding which are expected to shift the influence over research agendas from the researcher to different sets of external actors.

Firstly, if individual funding is for curiosity driven or “basic” science, usually provided by government agencies at different levels, or by private foundations allocated through competitive processes, we expect the jurisdiction of researchers to remain in the professional authority sphere and to be expressed collectively in the scientific community through the allocation of resources based on peer review.

Secondly, if third party funding is provided under industrial or services research contracts, the influence of the commercial or industrial interest in shaping research agendas and goals will increase. Nevertheless, indirectly, industrial funding that is brought to the organisation by the researcher through a professional-client relationship might influence his/her autonomy from the managerial authority, especially if the client’s feedback produces evidence of the researcher’s competence and value.

Thirdly, if individual funding is related to applied research, public mission-oriented research or is instrumented through priority-targeted public funding, then the external actors’ influence over the research agendas will be shared between policymakers in government agencies and the scientific community usually in charge of the peer review process. Bleiklie et al. (2015) have proposed the term “penetrated hierarchies” to label this interaction between intra-organisational authority or control dynamics and the influence of external actors^10^. In the next section we will argue that these general relations are mediated by the structural attributes of the different research configurations.

### Authority Sharing in Different Research Organisations and the Role of Funding

Research structures are contingent on many factors and there is not a dominant or universal model of research organisation; the potential impact of the funding patterns of research, either of researchers or organisations, varies in different conditions and contexts and could be affected by national or sectoral diversity.

In most of the previous studies of the organisation of research empirical analyses have not addressed the organisational level as it merits; although some of them refer to “labs or institutes” the empirical objects have most often been research teams (e.g. Pelz and Andrews 1966/76; Joly and Mangematin 1996; Larédo and Mustar 2000), rather than formal organisations. However, there have been some efforts at classification to combine the different research activities (basic, applied and experimental) with the diverse institutional sectors (Cole 1979).

Despite the historical account of eight different types of research laboratories (Van Rooij 2011), in recent descriptive literature (e.g. Arnold et al. 2010; OECD 2011) it has become traditional to identify three empirical categories of Public Research Institutes: government laboratories, academic institutes, and research and technology organisations. The rationale of this classification is related principally to the relevance of the empirical groups and self-identification. This classification of types combines elements of history, evolution and current attributes, but the assessment is mainly based on the dominant existing categories in a very limited number of countries and the stretching of the type’s labels.

A conceptual attempt to construct empirical taxonomies of research organisations was developed by Crow and Bozeman (1998); their objective was to characterise the type of science and technology products through the identification of the relative level of influence of the market versus the government. Cruz-Castro et al. (2012) empirically applied these ideas and examined the different sources of funding to classify two populations of research institutes.

More analytically grounded, the research of Cruz-Castro et al. (2015) or Sanz-Menéndez et al. (2011) identified two attributes of research organisations likely to condition research agendas: (a) the degree of external autonomy and resource dependence of the organisation—in terms of funding, human resources, access to external knowledge, for instance—and the associated degree of autonomy and discretion over resources; (b) the type of internal authority structure characterising the functioning of the organisation, and more precisely the relationship between centres’ researchers and management. Based on these dimensions, they constructed a typology of research organisations in correspondence with some empirical cases.

Instead of focusing on the empirical classes of Public Research Organisations we take a different approach: we concentrate on internal authority structures (see Table 2) and, instead of introducing the funding of organisations and researchers’ activities within the typology, we consider it as an exogenous factor (of a dynamic nature) which may reinforce or challenge internal authority balances.

**Table 2:** Internal authority sharing in different types of public research organisations

Key dimensions	Research organisation types as professional organisations		
	*Autonomous* Research Organisations	*Heteronomous* Research Organisations	*Managed* Research Organisations
*Research agenda choices*			
Strategic autonomy	Low for management/high for researchers	High for management/low for researchers	High for management/medium for researchers
Operational autonomy	High for researchers	Medium for researchers	High for project PI and medium for researchers
*Employment relations*			
Control over hiring	Community of researchers	Management	Joint between management and PI
Career and promotion	Tenure based-seniority based hierarchy	Based on contribution to organisational goals	Strongly performance-based
*Evaluation of tasks*			
Establishment of standards and performance evaluation	Externally established: Community of researchers	Internally established: management	Externally established, internally implemented
*Management*			
Management type	Hierarchical but weak, penetrated by researchers	Hierarchical, strong, research background	Hierarchical, dual, mixing administrators and researchers
Managerial discretion over collective resources (block grant funding)	Limited	High	High
Researchers’ discretion over individual project and contract funding	High	Low	High to medium for project leaders and PI

We have identified some dimensions in which we expect variations among the different types of research organisation. The first dimension, research agenda choices, relates to the types of autonomy and who decides the establishment of the research agenda and directions. We have termed this strategic autonomy; the related dimension of the discretion to decide over methods, techniques and work processes is what we have described as operational autonomy. The second dimension, employment relations, is concerned with who controls hiring and promotion, and how careers and promotion are related to the organisation. Thirdly, the evaluation of tasks refers to who controls the competences to establish performance standards and to evaluate such performance. Finally, there are three attributes related specifically to the management of the organisation: the relative demarcation between research and managerial responsibilities, the potential integration of the two and the relative strength of the latter; the discretion of managers over the use of infrastructure and other collective resources; and the discretion of researchers over the use of individual project and contract funding.

We now discuss, for each of the three organisational research types, the different dimensions selected and their relations with authority sharing and elaborate on the diverse funding forms and explore their expected effects.

#### Autonomous Research Organisations

In Autonomous Research Organisations the decisions regarding what lines of research to pursue as well as the control of the research process lies mainly with the researchers and research groups, who enjoy a high degree of strategic autonomy. Scientific standards are established externally by the professional or scientific community who control the training of new scientists in standardised sets of skills and bodies of knowledge as well as socialisation in shared values. Hiring is structured around scientific committees dominated by and even exclusively composed of researchers. Career rewards and promotions are allocated mainly on the basis of the perceived contribution of individual candidates to the field in committee-based processes. In autonomous research organisations, the dominant professional group of scientists will organise itself to evaluate the performance of its members through peer group controls.

Although these bottom-heavy organisations may display a degree of formal hierarchy, top-down coordination and control are very weak. Because of asymmetries of information and expertise, it is difficult for the management of this type of organisation to formulate a coherent, organisation-wide strategy, at least not independently of researchers. The dominance of the professional authority in autonomous research configurations is further enhanced if administrative management control over collective resources is limited. This limitation may have two sources: firstly, the formal delegation of the control of scientific infrastructure and other collectives to scientific directors and researchers; secondly, the existence of individual discretion over funding resources obtained externally by researchers themselves, from individual projects or contracts.

There has been great debate concerning the possibility that loosely coupled knowledge-intensive organisations of this type could be transformed into more “complete” organisations characterised by identity, hierarchy and rationality, as identified by Brunsson and Sahlin-Andersson (2000). Empirical research has focused principally on universities and not on public research institutes^11^.

To explore how funding may influence autonomy in this type of research organisations in a dynamic way, it is useful to distinguish between the types of funding described in the section *“Funding Resources and Control in Research Organisations”*. Considering organisational funding first, we believe that increases in earmarked block grant funding are not likely to affect the existing balance of authority and the dominance of researchers typical in this type of configuration. The seniority and tenure base structure of researchers’ careers in autonomous organisations makes changes in earmarked funding likely to be regularly distributed among the existing expenditure categories.

However, the situation might be different if we consider discretionary block grant funding at the disposal of leadership. Larger shares of discretionary block grant funding in autonomous research organisations are likely to improve the position of managers with respect to researchers, because the former could allocate and employ such resources to promote the strategic aims of the organisation; this allocation could take the form of new positions or contracts, internal funding projects^12^ or even the creation of new units with top-down appointed directors. In this way, leadership will have greater influence over research agendas via its increased capacity to select and hire researchers aligned with organisational strategic choices. Another way for management to balance its position *vis à vis* researchers and to negotiate the strategic aims of the organisation is through the type of external funding that comes from performance based funding systems.

In general, increases in individual project funding in autonomous organisations will reinforce the position of researchers *vis à vis* managers and the autonomy of the former in pursuing their research interests. Additionally, when individual researchers succeed in obtaining external funding, they may not only maintain or increase their autonomy from managers but also gain leverage to negotiate additional resources such as academic positions or institutional funding to support their agendas, feeding cumulative advantage processes. But even in autonomous research settings, where structure places considerable control and discretion in the hands of researchers, larger shares of external competitive individual project funding will also reinforce the foundations of the “republic of science”, including the reputational competition, with the consequent shift to the influence of scientific elites over research priorities. The extent to which increases in individual project funding will produce careers which are more autonomous, will also depend on who effectively controls the tenure system typical of this type of research organisation.

Different forms of individual project funding will also shape the sharing of authority: while curiosity-driven project funding will reinforce the authority of researchers, priority set and oriented public research funding or industry funding will provide external actors with more influence over research agendas.

#### Heteronomous Research Organisations

Heteronomous Research Organisations have specific goals established as organisational objectives and their management is capable of establishing a top-down organisational strategy to fulfil those goals. To accomplish this, this type of research organisation possesses structural devices that assure coordination and control of the individual contributions, and management is composed of professional administrators empowered with administrative authority to hire new entrants, allocate rewards and make decisions on promotions based on contributions to organisational goals. Projects or parts of projects are assigned to researchers, who have little autonomy to choose and at best medium discretion over project or contract funds. High internal strategic autonomy of managers coexists with substantial operational professional researchers’ discretion over methods and work processes, in the context of high standardisation of project development, and performance standards established internally by the organisation.

If researchers are involved in hiring and promotion decisions, it is through co-optation to participate in administrative committees. Internal mobility between administration and research is common, since there are no sharp distinctions between the two spheres of action, and promotion involves moving from the performance of research to research administration. Management in heteronomous organisations is not only stronger than in their autonomous counterparts, but proportionally more numerous. Since managers should be qualified as professionals themselves, this stronger hierarchy does not necessarily mean closer supervision of a bureaucratic type.

Perhaps one of the most important differences between autonomous and heteronomous ways of “organising” research relates to managerial discretion over collective resources (scientific and technical infrastructure and facilities, among others). The greater discretionary power of managers’ may be derived from two sources: firstly, heteronomous organisations may be strongly orientated to the client or user, or to the mandate of the principal, depending on their sector of operation; this means that greater control over financial costs and investment is exercised. Vertical accountability and reporting makes the delegation of decision-making rights over the use of costly equipment and other resources rather unusual. Another source of centralisation of discretion over collective resources stems from the need to invest managers with authority to prioritise projects according to overall strategic planning.

Considering how funding dynamics may affect heteronomous organisations, we expect that increases in earmarked block grant funding are unlikely to affect the existing balance of authority and the dominance of managerial authority. The case of discretionary research block funding is once more different, especially when it is instrumented through programme funding, which is not only likely to further reinforce managers’ authority with respect to researchers, but also to increase managerial accountability and therefore the influence of external sponsors over heteronomous Organisations which are oriented to specific goals.

In principle, if we ignore distinct structural characteristics, increases in individual project and contract funding will favour the internal position of researchers, but considering the minimal discretion that researchers have over this type of funding in heteronomous organisations, the greatest effect is related to the influence of external funders on research agendas, either public agencies through priority-oriented government funding or industry through contract research funds. The involvement of the scientific community in the research priority setting and the review process will increase its influence, if its participation in those processes is the norm.

#### Managed Research Organisations

In managed research organisations researchers and administrators are more or less equal regarding the authority they hold and the importance of their functional areas, and they coexist in a state of interdependence. Here, the distinction is not between strategy and operation as in the heteronomous type, but between organisational strategy and research project development. Macro issues related to the funding of the organisation, contract agreements with patrons and sponsors, long-term general lines of research, fund raising through patenting, licensing or product development, and top level recruitment, concentrate the leadership agenda. In such issues, the degree of strategic autonomy of managers is very high.

Organisational and research authority tend to overlap in this type of configuration, in which the top managerial positions are held by reputed directors with relevant scientific or technological reputation and performance. The integration of administrative and scientific authority could solve some of the collective action problems typical of research organisations and help to integrate organisational and individual goals in a coherent way.

Research groups, organised around a group leader or principal investigator (PI), may be allowed to function relatively autonomously over the life course of a project, from proposal to output. We expect the strategic autonomy of researchers in this type of structure to be intermediate, because they are recruited and hired to work on specific research projects in line with the long-term strategy of the organisation; in this respect, it is unlikely that they are free to radically change their agendas; their autonomy in the context of the research projects they lead or work on is expected to be very broad and their operational autonomy very high. As a consequence, the advancement of knowledge in one area or programme could contribute to the reshuffling of the objectives of the entire organisation. Here, researchers are hired to fulfil the content of a research programme, and create their own roles.

Research management resembles a figure of concentric circles in which the technical core or inner circle is projects, and this circle is dominated by scientists, but within the constraints established by the second circle in which the general management establishes expectations about performance and is responsible for conducting its evaluation. The coordination of the use of collective resources, including infrastructure and the allocation of internal funding and positions, is also in this second circle, but the control of hiring and careers is likely to be a function shared between research administrators and PI researchers. The scientific reputation principle based on performance is expected to work strictly in this type of organisation, with researchers below the group leader level enjoying considerably less autonomy.

This type of organisation has been sometimes referred to in the literature as “hybrid” (Diefenbach and Sillince 2011) or “post bureaucratic” organisations (Clegg 2012) in which one of the original ideas was to reduce formal hierarchy via the introduction of teams and projects^13^. In the literature on the public sector, they are presented as organisational innovations or developments associated to escaping from the rigidities of bureaucratic structures, but their effects could be contradictory. Additionally, organisations of this type have an evolutionary character, and they could have specific national characters and show singular path dependence features.

Comparatively, it can be said that managers are structurally more powerful in managed organisations than in autonomous ones, and that researchers are more autonomous in managed organisations than in heteronomous ones, unless they are far below the project leadership. The delegation of a part of strategic autonomy to researchers in managed structures comes at the price of strong performance evaluation, which is a shared central organisational function and follows standards established externally by the profession but internally implemented by the centre, with levels of demand that vary depending on the reputational or market position of the organisation in the field, among other factors.

This type of configuration might involve a complex but stable equilibrium between the professional and bureaucratic authority. Turning to funding dynamics, it is to be expected that increases in discretionary block grant funding in managed organisations, especially if they are instrumented through “performance based funding” will reinforce the standing of the organisational leadership with respect to researchers. In the context of the strongly performance-based management typical of this configuration, these augmented discretionary resources could be used by managers to attract researchers to the strategic visions adopted by the organisation by allocating incentives and rewards, or simply by giving greater internal resources to specific projects.

The consideration of individual project or contract funding in managed research organisations needs to take into account social hierarchy properties and the prominent role of the project leadership layer. In principle, more individual project funding is not likely to change the more or less balanced sharing of internal authority between PI and managers. This stability is aided by the stratified research structure (centred on PI group leaders with a management style that develops recruitment and promotion practices consistent with the values of the lead researchers) typical of this type of organisation. In any case, as in the other two structural types, increases in project and contract funding are likely to make external actors more influential, with the possible exception of curiosity-driven project funding.

## Final Remarks

Research organisations are not equally wealthy, and even regardless of their general wealth, they may be affected by funding cycles or crises over concrete periods of time. The general level of resource endowment of organisations is expected to affect the interaction between funding and structure. Managers in autonomous organisations are structurally less powerful than in the other two types, but levels and types of research funding can mediate this balance. Low levels of block grant or organisational funding make autonomous organisations resource dependent, and their management reliant on the research interests of the principal investigators who apply for external funds and which, in aggregate, largely determine the scientific profile of the organisation; in these circumstances, managers’ already low strategic autonomy in this type of entities for structural reasons is likely to be further reduced, unless they can expand control over scientific recruitment or over common collective infrastructure through increases in discretionary funding.

The structurally higher internal authority of researchers versus managers in autonomous organisations may or may not lead to high levels of real autonomy in “poorly endowed” research organisations; here researchers’ pressing need for individual grants to perform research activities will in turn allow for an increasing influence of external actors on researchers’ agendas, either scientific elites (in the case of peer reviewed basic science funding), policymakers and the research community (in the case of mission-oriented or prioritised funding) or industrial interests (in the case of industrial or contract funding).

Structurally, managers in heteronomous and in managed research organisations have higher shares of authority in relation to their researchers and therefore a higher level of influence over research orientation *vis à vis* researchers in autonomous organisations. Once more, this balance is likely to be mediated by the general wealth of the organisation. Large and continual amounts of block grant funding (especially if its use is discretionary) involve a sizeable endowment of resources from funders who delegate authority over these funds to directors, based on trust and expectations; this is likely to make managers and directors in wealthy heteronomous research organisations and their managed counterparts strong and very influential concerning the research agendas of their organisations (Gläser et al. 2014), although more accountable to the external funders.

But the opposite does not necessarily hold true, as researchers do not become more powerful in poorly endowed heteronomous and managed organisations; the need to search for additional income streams from competitive projects or private contracts may strengthen the competition for resources, but it may or may not increase the authority and discretion of principal investigators (not all researchers) versus management. Again, what is much more likely is that this higher resource dependency will lead to an increased influence of external actors on internal agendas. It is important to note, therefore, that in a context of funding pressures, managers might face a dilemma: while they may see the acquisition of funding by researchers as a challenge to their authority, they may have to encourage such funding if the organisation is pressed, for instance, by government, to develop third party funding.

This paper has addressed the question of how variations in public research organisations are related to various dimensions of the autonomy and control of researchers over research objectives and content. We have argued that there are at least two sources of legitimate authority within research organisations, one derived from hierarchy (bureaucratic) and another derived from the community (professional), and that while the share of authority between researchers and managers over research goals is essentially structural, it is empirically mediated by the dominant funding portfolio of organisations and the corresponding endowment of resources at the disposal of directors or researchers.

Inspired by the organisation theory, we have proposed an analytical typology of research configurations based on several dimensions that account for the different internal authority balance between researchers and the organisational leadership; we believe this might be useful as a guide for the empirical analysis of diversity in the field of public and semi-public research institutes. Rather than assuming that ways of funding have direct and determinant effects over the research agendas and goals of researchers, we consider funding as one condition of research production among others. Instead of introducing the funding of the organisation and of researchers within the typology, we consider it to be an exogenous factor (of a dynamic nature) that may reinforce or challenge internal authority balances. In this way, we have been able to advance various propositions emerging from changing dynamics in funding levels or types. It should be noted that the shift in authority relations should not be seen as a purely zero sum game. As critically noted by Kehm (2013), what organisations win through their managers is not an automatic loss for researchers, since it is necessary to keep in mind the role of scientific elites and other external actors in the funding processes.

Although by their very nature the types within typologies seem static and homogenous, we nevertheless believe that our approach allows certain processes and internal diversity to be captured. Depending on their varying ability to attract external funding, individual researchers or departments within the same research organisation might have different degrees of autonomy from managers, resulting in increasing internal competition and even fragmentation. Although we acknowledge that tensions, conflicts and managerial dilemmas are likely to develop in research organisations, we have not addressed here how this might led to organisational change.

In this direction, a future line of inquiry could address the effects of social structures on organisational forms (Stinchcombe 1965). We have addressed the hypothetical effects of funding changes on internal authority sharing within research organisations; future complementary work should tackle the emergence of variance in the Public Research Organisation types that may result from equilibrium disturbances in the social structures surrounding organisations.

We have tried to avoid a normative approach and have focused on plausible analytical associations. We have concentrated on advancing some general propositions and offering some broad avenues for empirical testing. Some factors have been held constant, the most important of these being research fields. It should be acknowledged, however, that there may be some field-specific factors related to the relative importance of particular resources and infrastructure for the conduct of research which have not been addressed here. Research fields are not equally resource dependent. They differ, among many others, in three aspects: firstly, their need for costly infrastructure and equipment; secondly, their human resources intensity; and thirdly, their time frame requirements for producing research outputs. Undoubtedly, these factors and their combination intervene in our proposed types. Research in some fields requires a volume and type of material and human resources and equipment which exceed the fundraising capacity of any individual researcher.

In principle, the expectation is that areas requiring high levels of funding and investment in scientific infrastructure and equipment make researchers less autonomous, as this type of resource is usually provided by the organisation and subject to control. Moreover, in fields where these high funding requirements are combined with long time periods required to produce results (most fundamental research fields, frontier or emerging basic fields), researchers will have to share more authority over research goals with other actors than otherwise. In such fields, individual funding could be additional and provide researchers with a certain discretion, but organisational funding would be an inescapable necessary condition, even in autonomous research organisations where the use of infrastructure might be delegated.

By contrast, in fields less demanding in terms of resources, and/or with already developed technologies/methodologies which allow clients or knowledge users to be reached (including industry, government agencies, the scientific community, etc.) in shorter times (for instance: policy studies, applied social science research, and some types of applied technology services), individual researchers are better placed to exercise their autonomy, provided they are capable of accessing external funding, even in the more restricted context of heteronomous or managed organisations. In these circumstances, researchers can take advantage of their individual fundraising capacity to advance their organisational careers.

The relative value of our analytical exercise will need to be tested through empirical case studies which also introduce scientific fields. We hope this is a step forward into the complex process of characterisation of the research sector and its dynamics.

## Footnotes

1 We provisionally define Public Research Organisations (PROs) as independent organisations which have R&D as their main activity and mission (thereby excluding universities) and they also receive significant influence from governments, through control, funding, mission definition or managerial appointments. For the OECD (2011) the non-university public research sector comprises: public and semi-public research organisations which conduct basic or applied research, technology and innovation services, and disseminate their results by way of training, publication and technology transfer, which could, regardless of their legal status, be publicly owned to different degrees, and/or funded significantly from public sources either via block grants, competitive funding or contract-based research, so as to gain public missions.

2 It must be acknowledged that this paper holds some factors constant, most importantly research fields.

3 It is interesting to recall the fashionable debates on the radical departures of the science system that the discussion on “Mode 1 and Mode 2 of knowledge production” (Gibbons et al. 1994) presented in the 1990s and the limited nature of empirical changes identified (Weingart 1997; Shinn 1999).

4 Greenwood et al. (1990) described the classical mode of organisation of professional service provision in the private sector and its changes in areas such as law, accountancy, consultancy, etc. They portrayed the professional partnership (P2) and the consolidation of a new archetype of “Managed Professional Business” (Cooper et al. 1996; Hining et al. 1999) with significant departures from previous models in control systems and in the ways in which structures deal with differentiation and integration; managed professional organisations resemble Scott’s conjoint model.

5 In this sense we attempt to describe organisational configurations that are formed from characteristics and elements of the given phenomenon but which are not meant to correspond to all of the characteristics of any one particular case.

6 We believe our framework could be applied to a wide variety of public and non-profit research organisations considered both as a whole or their subunits, provided that a level of managerial leadership could be identified.

7 Growth in R&D systems has emerged together with the creation or expansion of new types of research performing organisations, sometimes called “hybrids”, with specific structural attributes, probably more than with radical transformations of existing organisations (Cruz-Castro and Sanz-Menéndez 2007; Gulbrandsen 2011).

8 As suggested by Diefenbach and Sillince (2011: 1527), in hybrid organisations (Menard 2004), informal hierarchy (that is, relations of subordination that emerge from social interaction, the bases of which could be reputation, performance, etc.) complements formal hierarchy in areas where the latter cannot reach members effectively and will dominate the organisation.

9 We are not assuming that managers in all research organisations are interested in defining the research problems addressed by researchers. This is an empirical question which also depends on whether and how collective goals are established. Instead, we argue that certain types of funding empower the leadership to influence the research profile of the organisation more than other types. This influence has gained importance in the context of increased autonomy granted to public sector organisations along with augmented managerial accountability expected by funders.

10 More precisely, the penetration of hierarchical intra-organisational structures by horizontal intra-organisational networks in which the members of the research profession also participate. A case in point could be the operation of joint research organisations within universities, which result in jointly operated centres in which the influence of academic hierarchies may further undermine the relatively weak authority of managers.

11 With reference to universities, there is a significant amount of literature highlighting the limited actorhood of such organisations (Whitley 2010, 2012). It can be said that evidence on the effect of the reforms is mixed and focused only on universities; for example, Seeber et al. (2015) found that, despite heterogeneity and the existence of two distinct groups of more traditional or more managerial universities, both of them could hardly become complete organisations even when “modernisation” policies in line with NPM were strong. Instead of a replacement of values and practices, they argued that managerialism was not necessarily at odds with collegial influence on decision making and professional autonomy. In contrast, De Boer et al. (2007) found a “re-engineering” of Dutch universities as more complete or corporate actors. In that paper they provide an overview of some indicators operationalising the concepts of identity, hierarchy and rationality in an empirical case.

12 We refer to R&D project funding provided by organisations to their own researchers.

13 However, Diefenbach and Sillince (2011: 1526) argue that an unintended outcome of the team or project-based structure may be a duplication of hierarchy, and an increase of the social control of contributions and performances, often meaning greater pressure, and more informal means of control, instead of less. Likewise, Clegg (2012: 73–74) claims that this knowledge-intensive type of hybrid or post-bureaucratic organisations cultivate a culture of competition, ambition and a method of circulation through programmes and projects, with three likely outcomes: firstly, they differentiate sharply those likely to advance and those who will not; secondly, top management decisions (resource allocation, project termination, team leader promotion/dismissal) shape the chances of those participating in projects. Thirdly, project leadership becomes a complex elite layer to pass through but also a fast route to upward mobility in the organisation.
